# Endoscope-assisted single-port intragastric surgery for gastric submucosal tumors in the fornix

**DOI:** 10.1055/a-2846-4728

**Published:** 2026-05-21

**Authors:** Akihiro Takagi, Keisaku Yamada, Tsutomu Tanaka, Nobuhito Ito, Kazunari Misawa, Yuichi Ito, Masahiro Tajika

**Affiliations:** 1 Department of Endoscopy 538357 Aichi Cancer CenterNagoyaJapan; 2 Department of Gastrointestinal Surgery 538357 Aichi Cancer CenterNagoyaJapan

Resection of gastric submucosal tumors (SMTs) located in the fornix or the posterior wall remains technically challenging. Endoscopic full-thickness resection is limited by poor endoscope maneuverability and the difficulty of achieving secure defect closure 1 2 , while laparoscopic and endoscopic cooperative surgery often provides insufficient visualization in these anatomically restricted regions 3 . Intragastric surgery was developed as a minimally invasive technique that allows direct visualization within the gastric lumen 4 . Building upon this concept, we present a novel endoscope-assisted single-port intragastric surgery (EASIS) technique that enables full-thickness resection under simultaneous endoscopic and laparoscopic visualization.

**Fig. 1:**
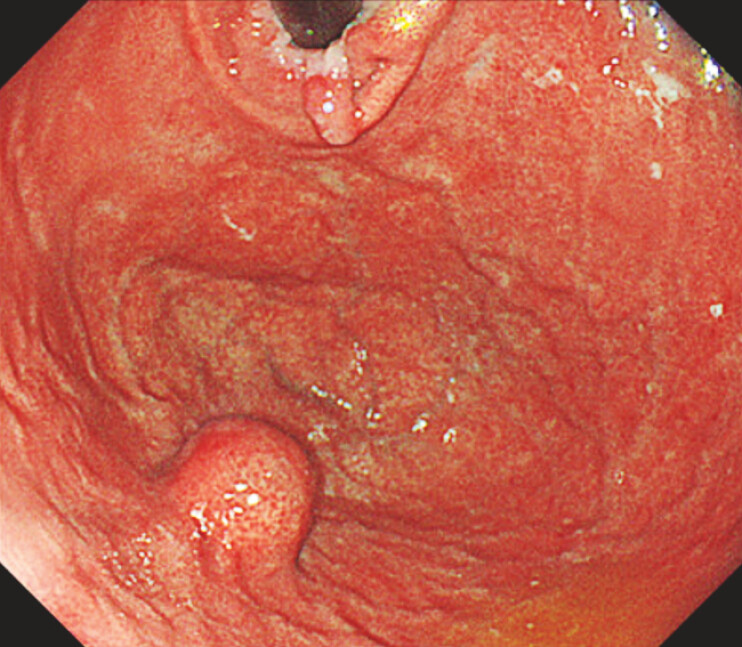
The lesion was a 15 mm submucosal tumor on the posterior wall of the fornix.

A 49-year-old man was referred for the treatment of a gastrointestinal stromal tumor (GIST) on the posterior wall of the fornix ( Fig. 1 ; Video 1 ).

Endoscope-assisted single-port intragastric surgery (EASIS).Video 1

**Fig. 2:**
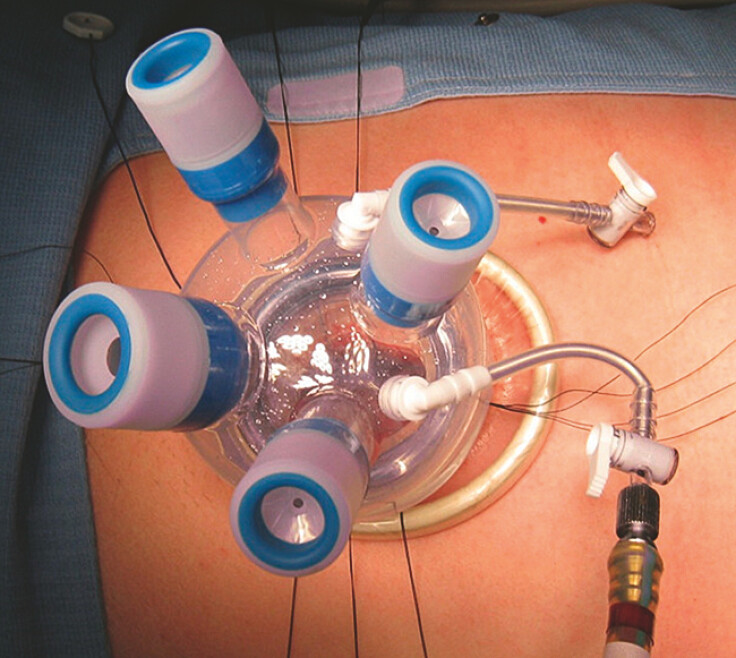
A multi-port device was placed through a supra-umbilical incision to establish intragastric access.

**Fig. 3:**
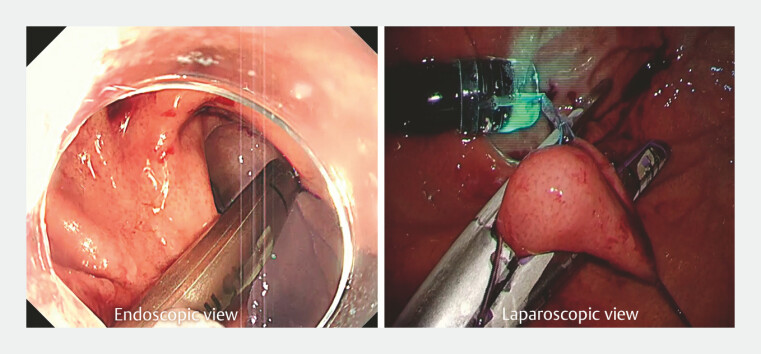
Simultaneous endoscopic and laparoscopic views during endoscope-assisted single-port intragastric surgery (EASIS), enabling precise resection.

Under general anesthesia, a 2-cm supra-umbilical incision was made, the lower gastric body was exteriorized, and a multi-port device was inserted to establish intragastric access ( Fig. 2 ). Two traction sutures were placed around the SMT, providing stable exposure. An oral endoscope was then advanced, and a detachable snare was positioned around the tumor via the traction sutures. After confirming adequate constriction, a linear stapler was introduced through the multi-port and used to transect the lesion at the base of the snare, achieving full-thickness resection without tumor spillage or peritoneal opening ( Fig. 3 ). Histopathological examination revealed a GIST measuring 17 × 13 × 11 mm with negative margins. The mitotic count was <5 per 50 high-power fields, and the Ki-67 labeling index was 1–2%, indicating low proliferative activity.

EASIS enables simultaneous endoscopic and laparoscopic visualization, allowing precise control and compensation for blind areas. Endoscopic traction minimizes instrument interference during single-port intragastric surgery. Because the gastric wall is not opened to the peritoneal cavity, the risk of tumor dissemination is minimized. This dual-view technique offers a feasible, minimally invasive, and oncologically safe approach for the resection of SMTs in difficult regions such as the gastric fornix and posterior wall.

Endoscopy_UCTN_Code_TTT_1AT_2AD
